# Supplementary material for: 1-Carbomethoxy-β-Carboline, Derived from Portulaca oleracea L., Ameliorates LPS-Mediated Inflammatory Response Associated with MAPK Signaling and Nuclear Translocation of NF-κB
Source: Molecules. 2019 Nov 7;24(22):4042. doi: 10.3390/molecules24224042 (PMC6891712; doi:10.3390/molecules24224042)
Supplement: Supplementary file 1 [file molecules-24-04042-s001.pdf]

Article

1-Carbomethoxy-β-Carboline, Derived from *Portulaca oleracea* L., Ameliorates LPS-Mediated Inflammatory Response Associated with MAPK Signaling and Nuclear Translocation of NF-κB

Kang-Hoon Kim [1,†], Eun-Jae Park [1,2,†], Hyun-Jae Jang [1], Seung-Jae Lee [1], Chan Sun Park [1], Bong-Sik Yun [2], Seung Woong Lee [1,*] and Mun-Chual Rho [1,*]

1 Immunoregulatory Material Research Center, Korea Research Institute of Biotechnology, 181 Ipsin-gil, Jeongeup-si, Jeonbuk 56212, Korea

2 Division of Biotechnology and Advanced Institute of Environment and Bioscience, College of Environmental and Bioresource Sciences, Chonbuk National University, Iksan-si, Republic of Korea

* Correspondence: lswdoc@kribb.re.kr (S.W.L.); rho-m@kribb.re.kr (M.-C.R.); +82-63-570-5264 (S.W.L.); Tel.: +82-63-570-5230 (M.-C.R.); Fax: +82-63-570-5239

† These authors equally contributed to this study.

	Content	Page
**Figure S1**	HRESIMS spectrum of compound **8**	2
**Figure S2**	IR spectrum of compound **8**	3
**Figure S3**	^{1}H NMR (600 MHz, methanol-d_4) spectrum of compound **8**	4
**Figure S4**	^{13}C NMR (150 MHz, methanol-d_4) spectrum of compound **8**	5
**Figure S5**	COSY (600 MHz, methanol-d_4) spectrum of compound **8**	6
**Figure S6**	HMQC (600 MHz, methanol-d_4) spectrum of compound **8**	7
**Figure S7**	HMBC (600 MHz, methanol-d_4) spectrum of compound **8**	8
**Figure S8**	^{1}H NMR (600 MHz, methanol-d_4) spectrum of compound **15**	9
**Figure S9**	^{13}C NMR (150 MHz, methanol-d_4) spectrum of compound **15**	10
**Figure S10**	COSY (600 MHz, methanol-d_4) spectrum of compound **15**	11
**Figure S11**	HMQC (600 MHz, methanol-d_4) spectrum of compound **15**	12
**Figure S12**	HMBC (600 MHz, methanol-d_4) spectrum of compound **15**	13
**Figure S13**	^{1}H NMR (600 MHz, methanol-d_4) spectrum of compound **20**	14
**Figure S14**	^{13}C NMR (150 MHz, methanol-d_4) spectrum of compound **20**	15

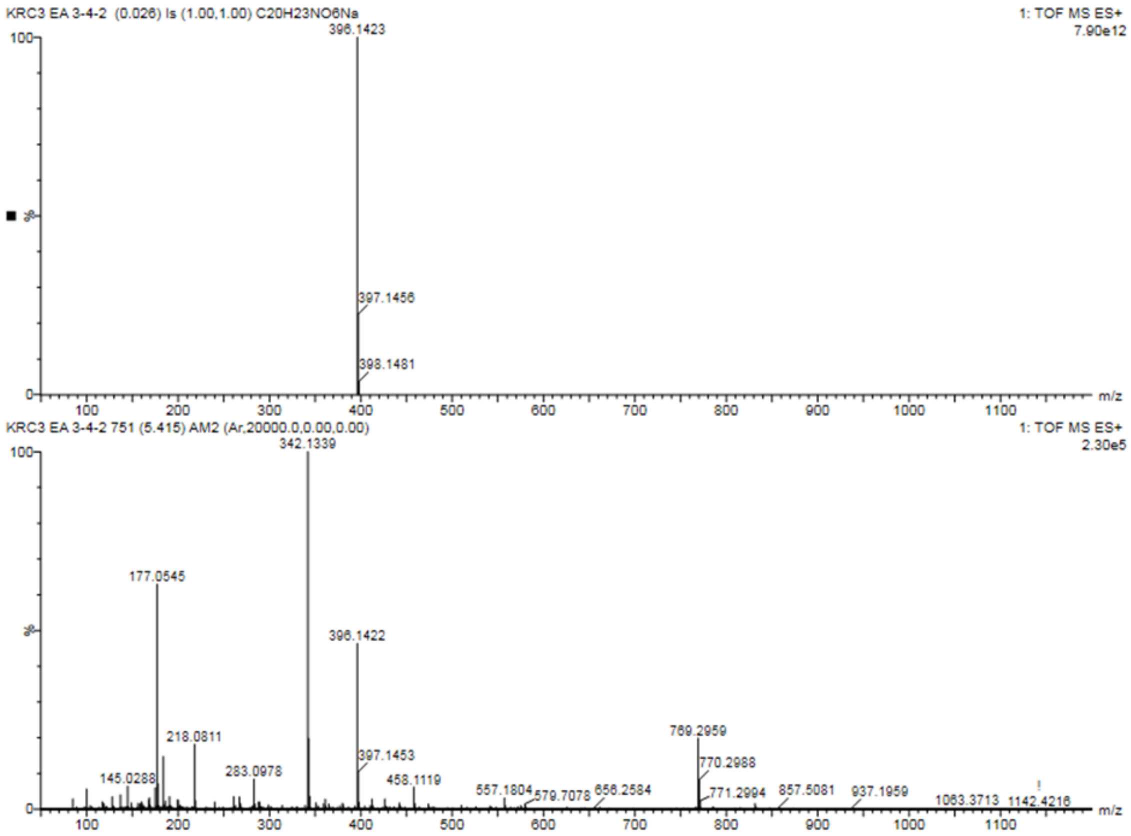

Figure S1. HRESIMS spectrum of compound **8**

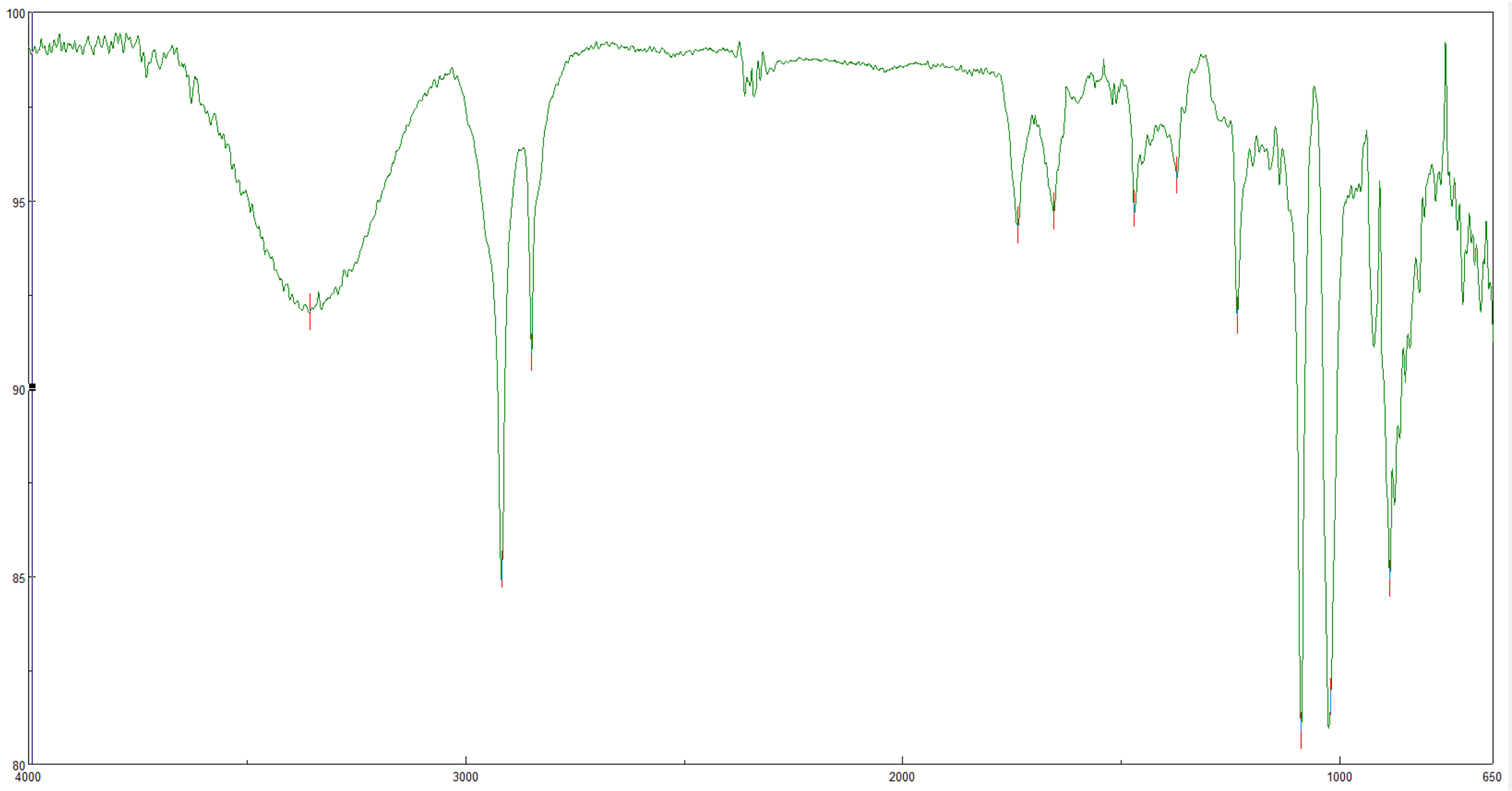

Figure S2. IR spectrum of compound **8**

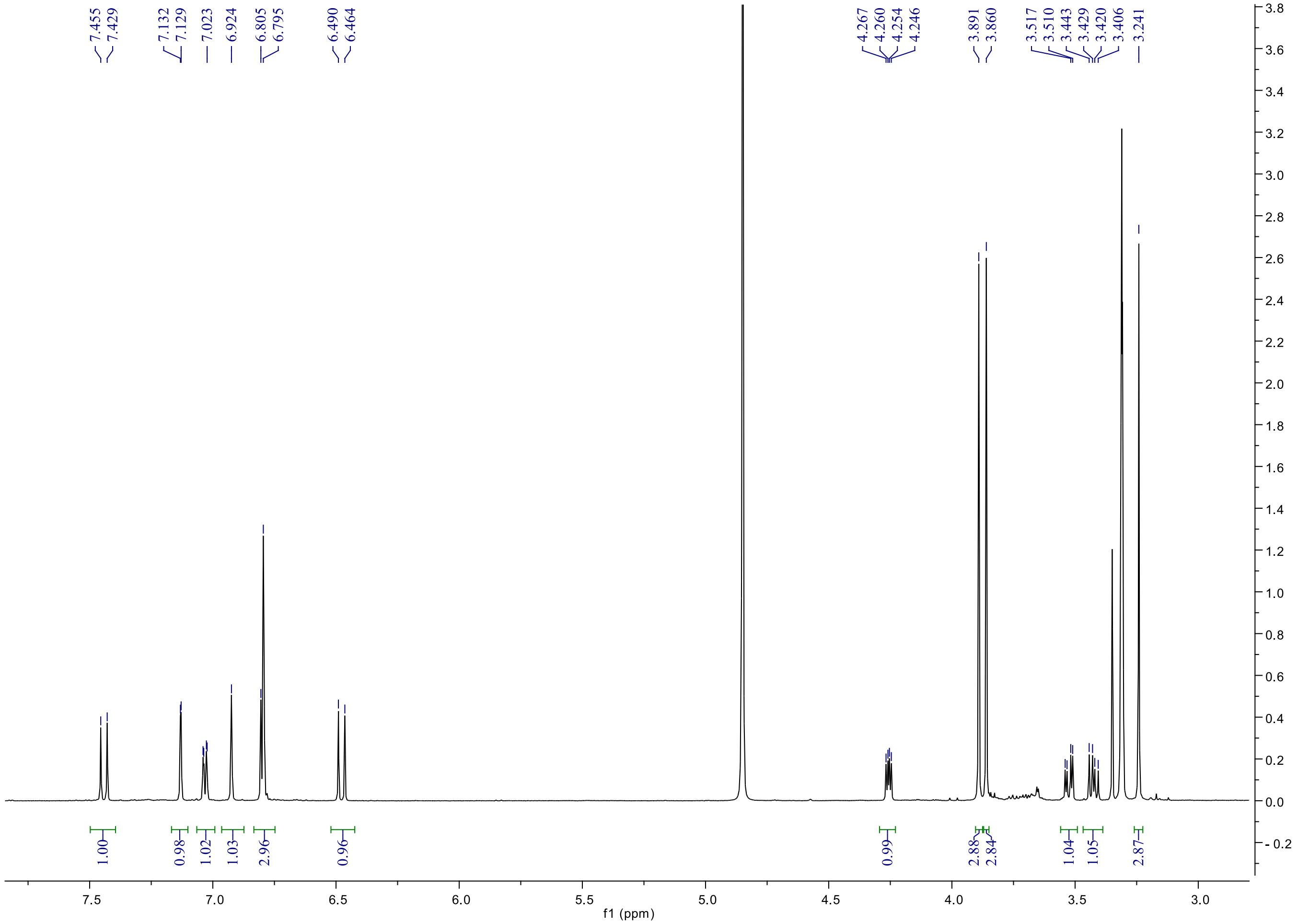

Figure S3. ¹H NMR (600 MHz, methanol-d_4) spectrum of compound **8**

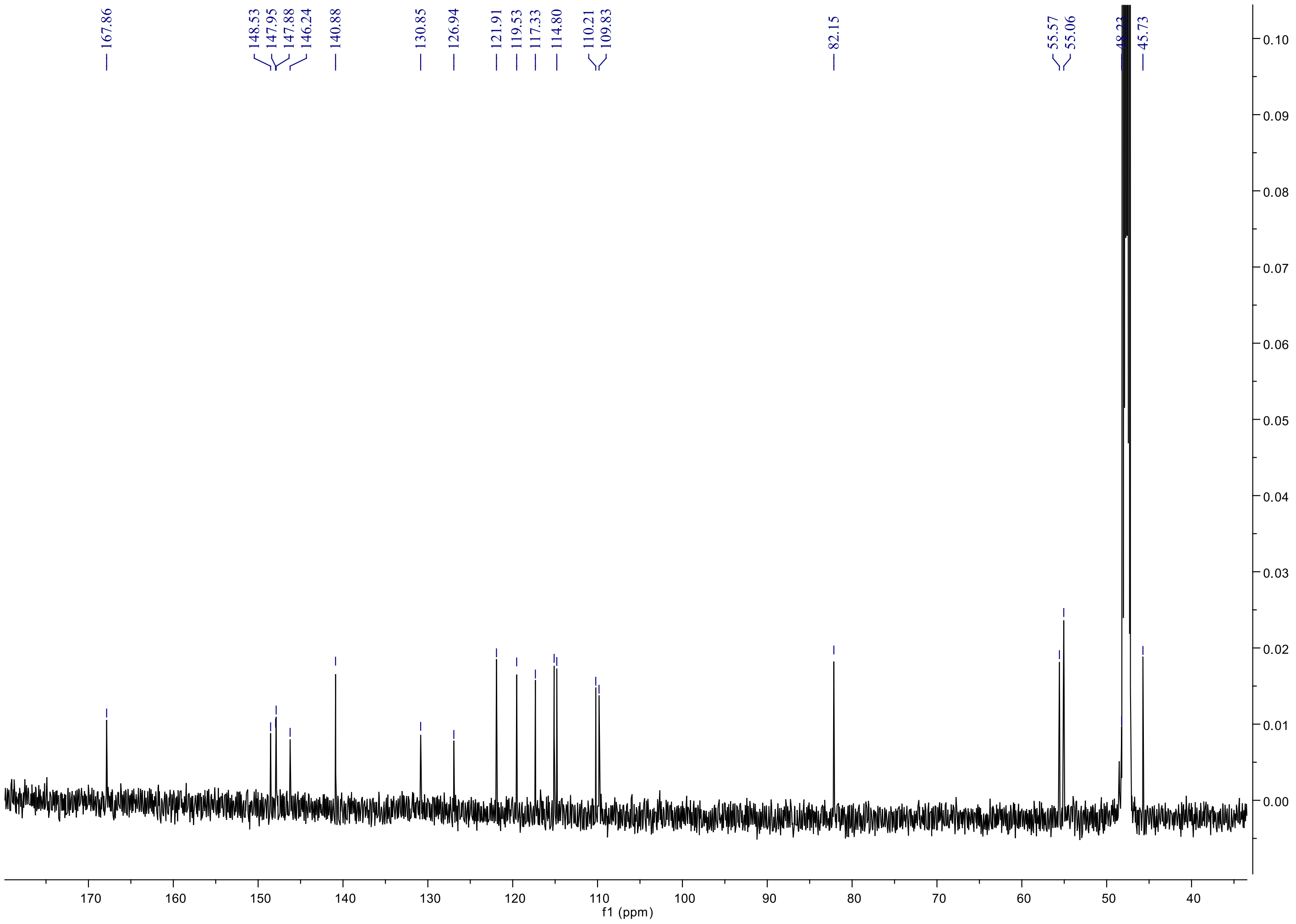

Figure S4. ^{13}C NMR (150 MHz, methanol-d_4) spectrum of compound **8**

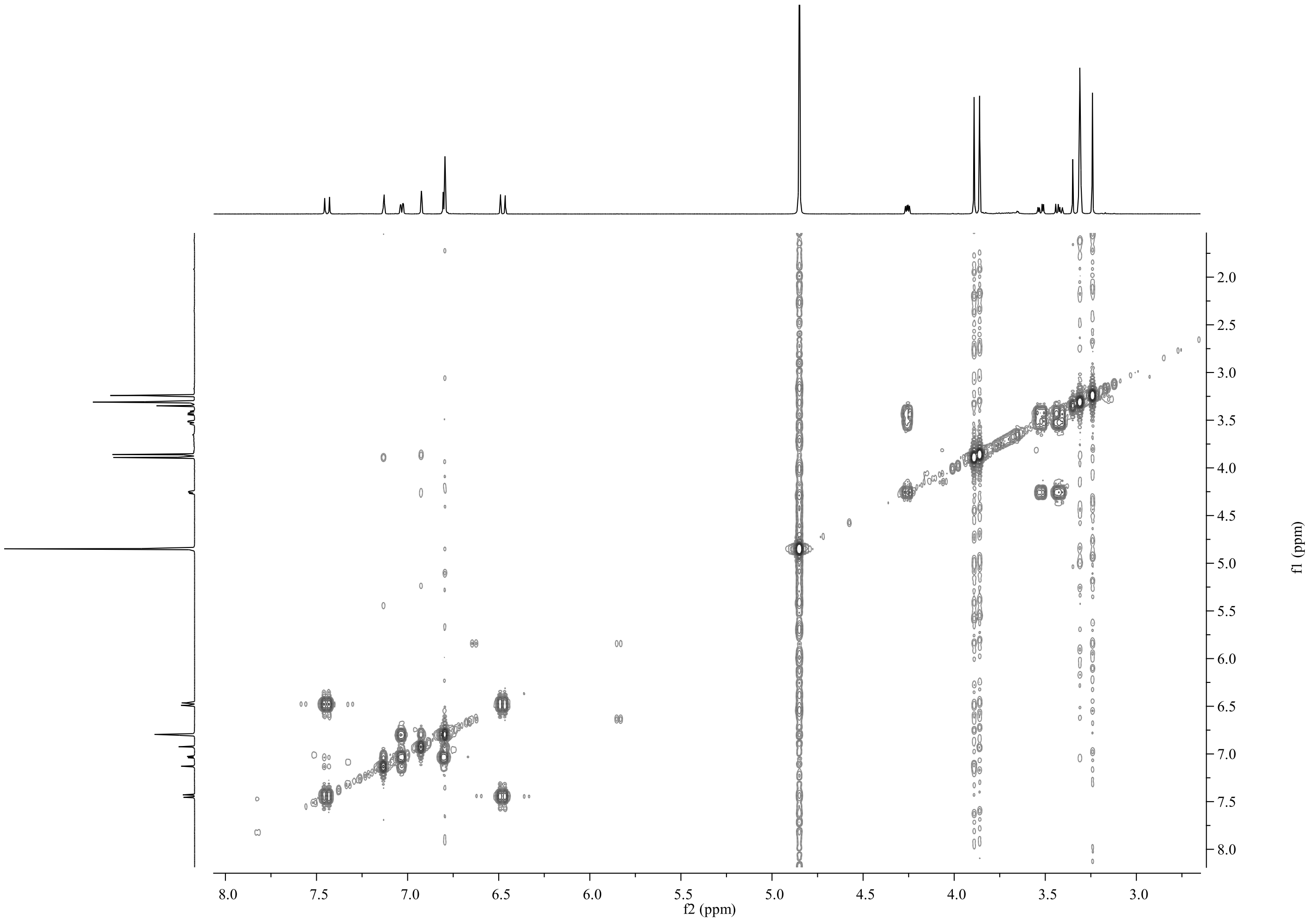

Figure 15. COSY (600 MHz, methanol-d_4) spectrum of compound **8**

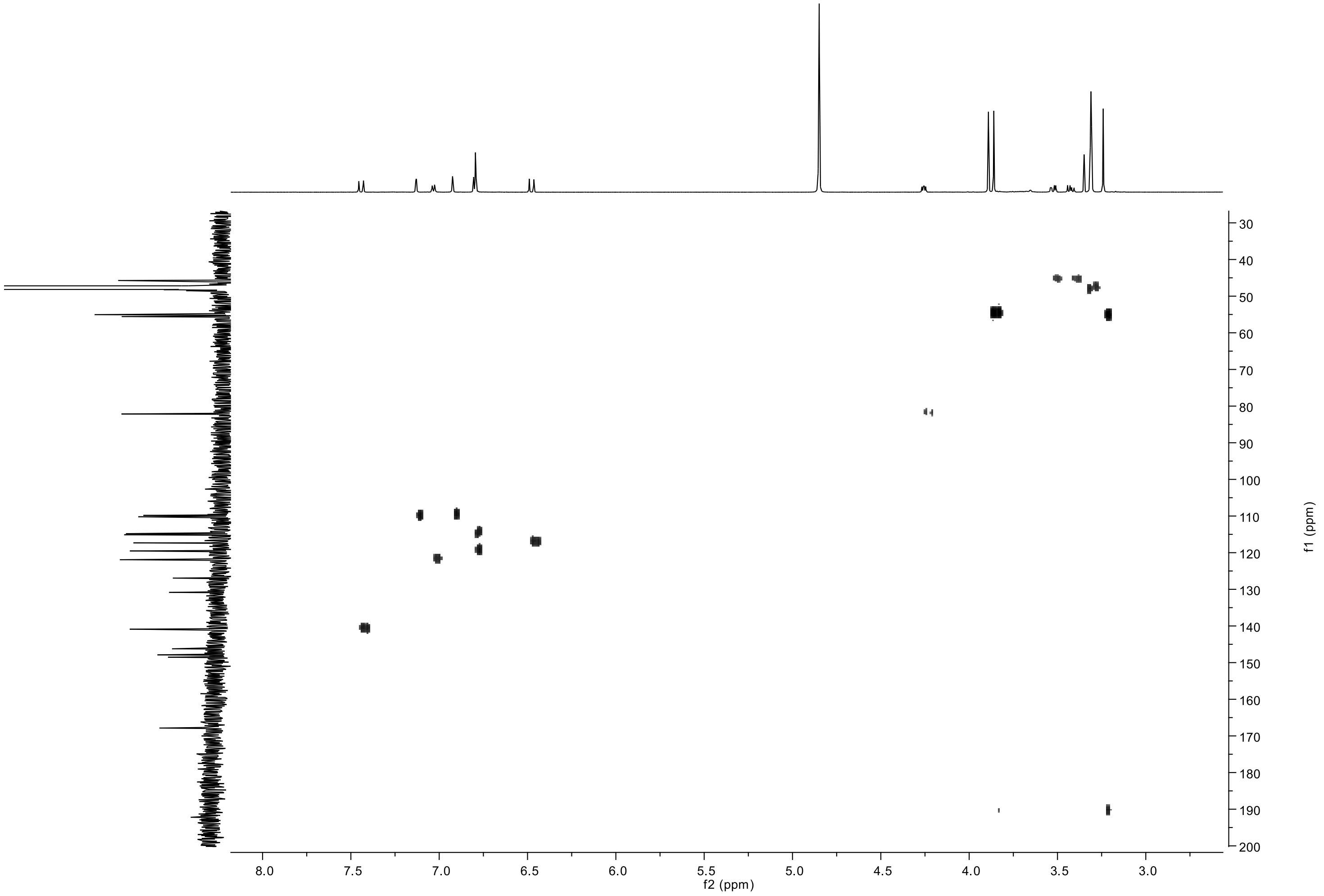

Figure S6. HMQC (600 MHz, methanol-d_4) spectrum of compound **8**

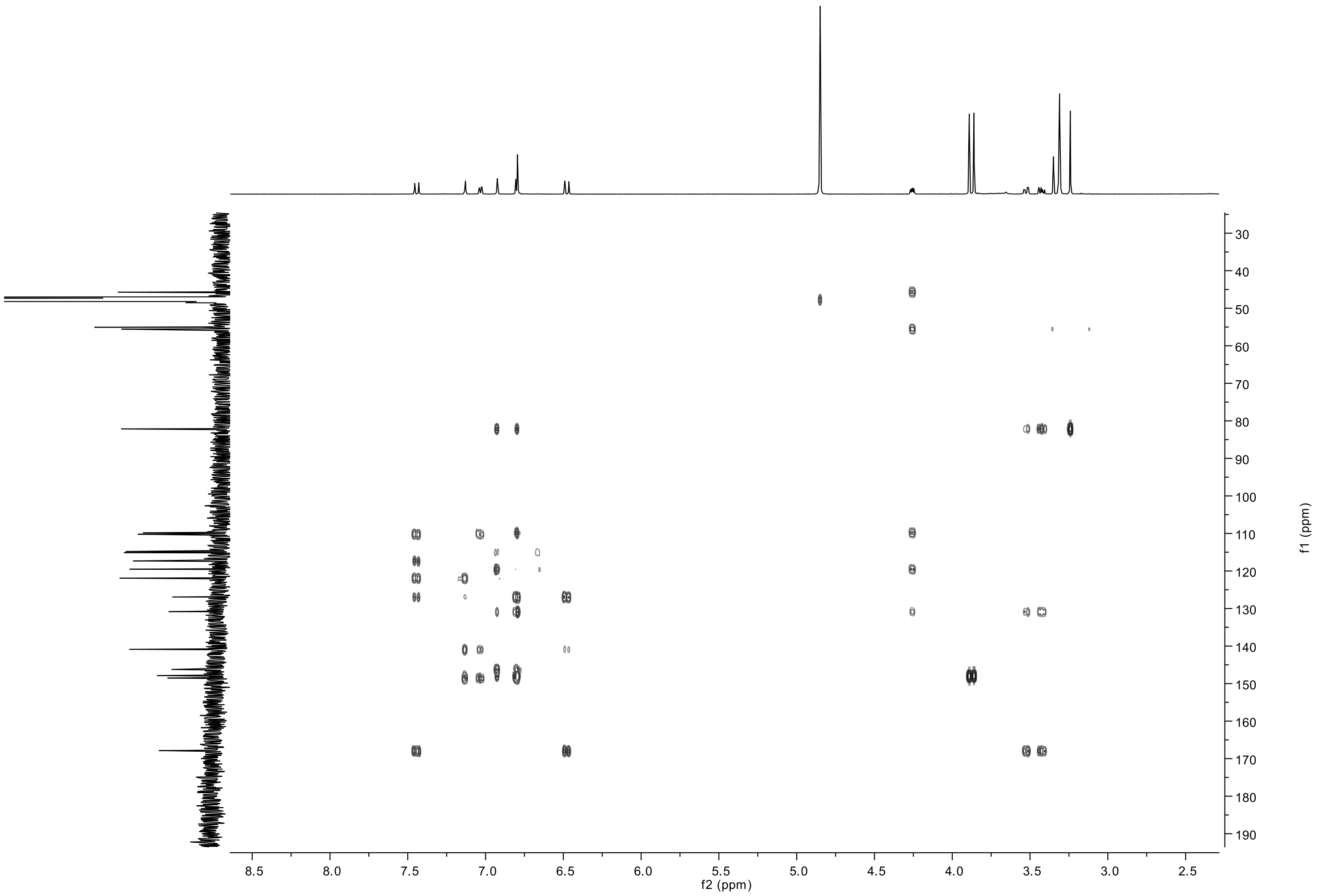

Figure S7. HMBC (600 MHz, methanol-d_4) spectrum of compound **8**

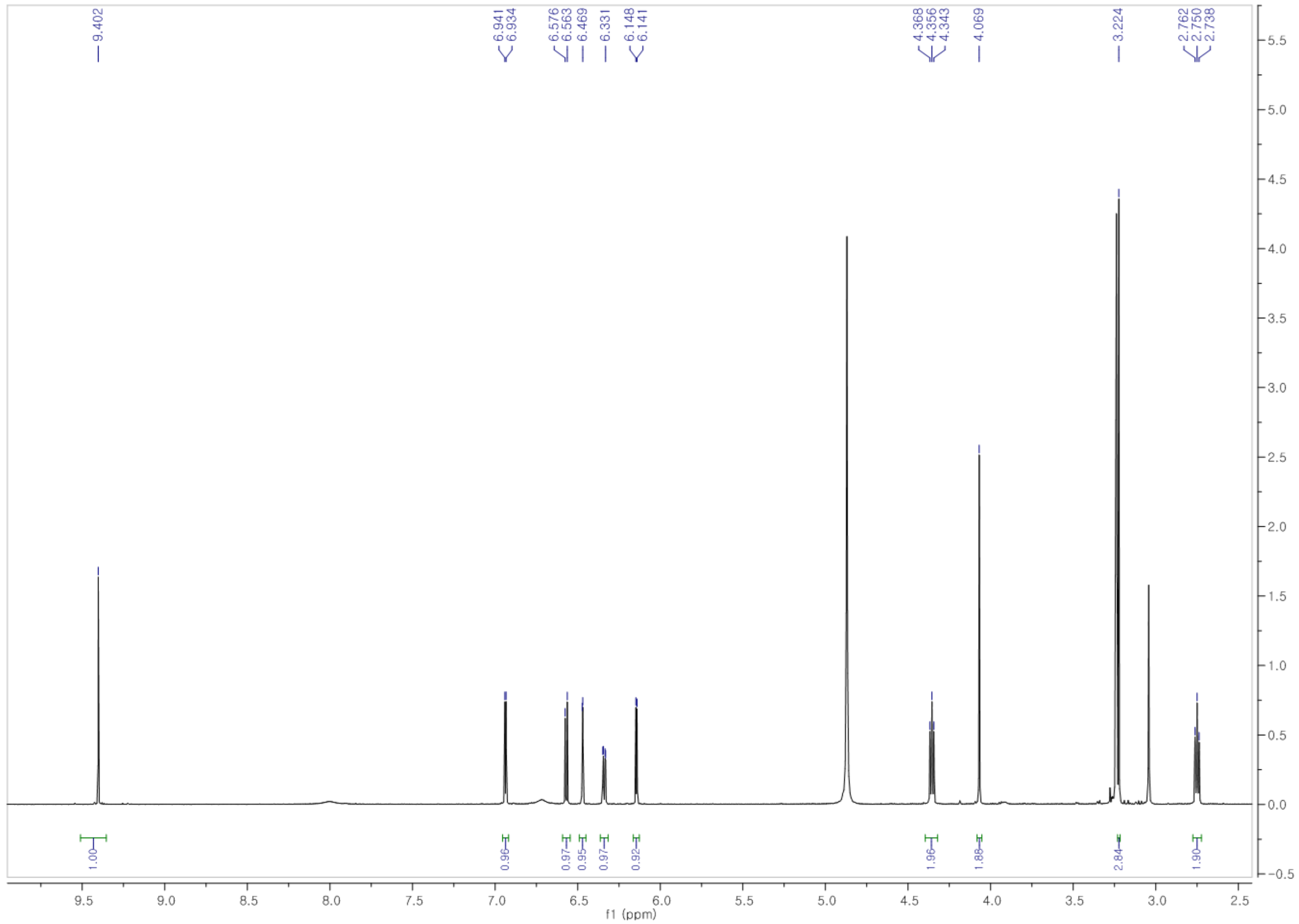

Figure S8. ^{1}H NMR (600 MHz, methanol-d_4) spectrum of compound **15**

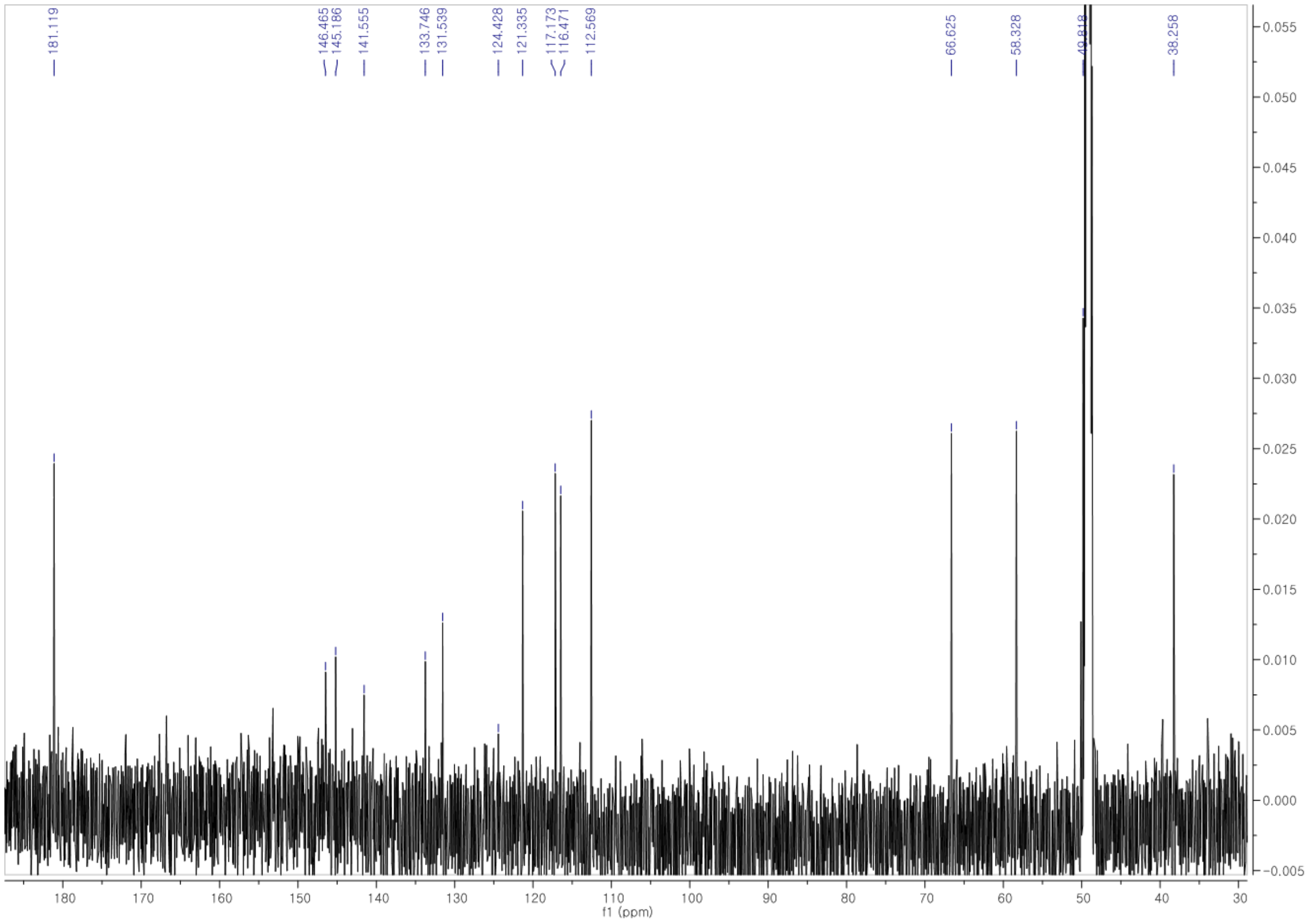

Figure S9. ^{13}C NMR (150 MHz, methanol-d_4) spectrum of compound **15**

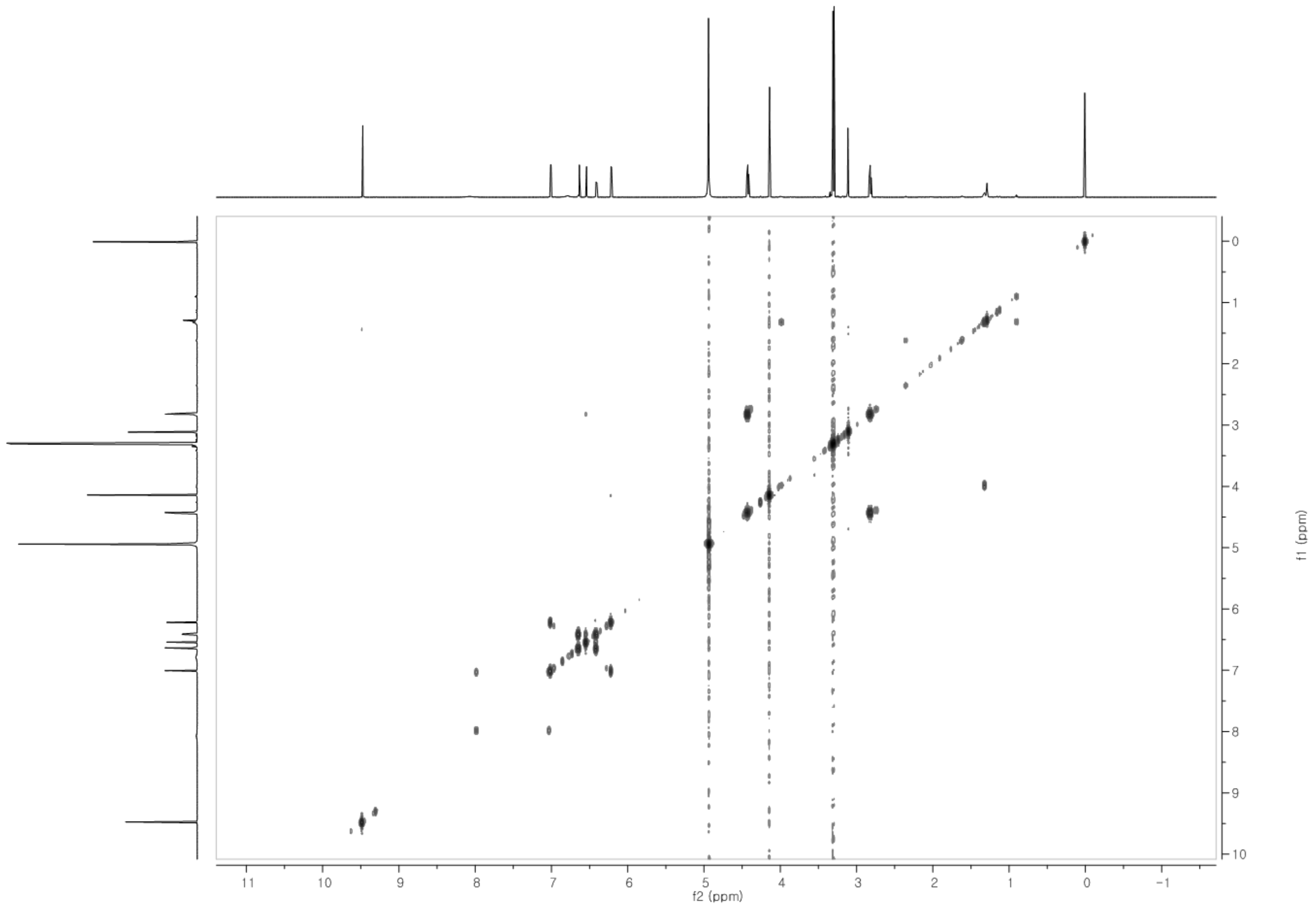

Figure S10. COSY (600 MHz, methanol-d_4) spectrum of compound **15**

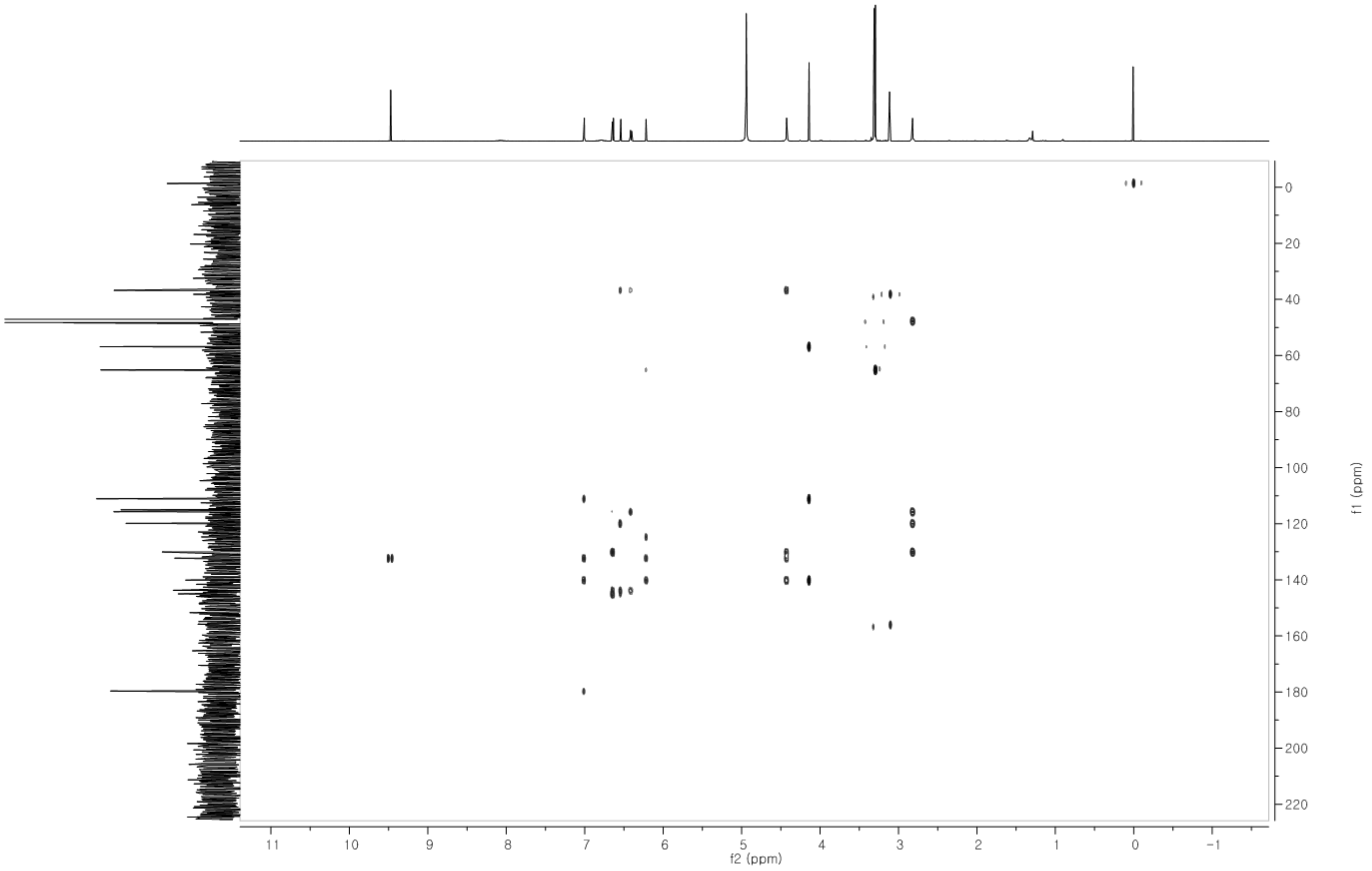

Figure S11. HMQC (600 MHz, methanol-d_4) spectrum of compound **15**

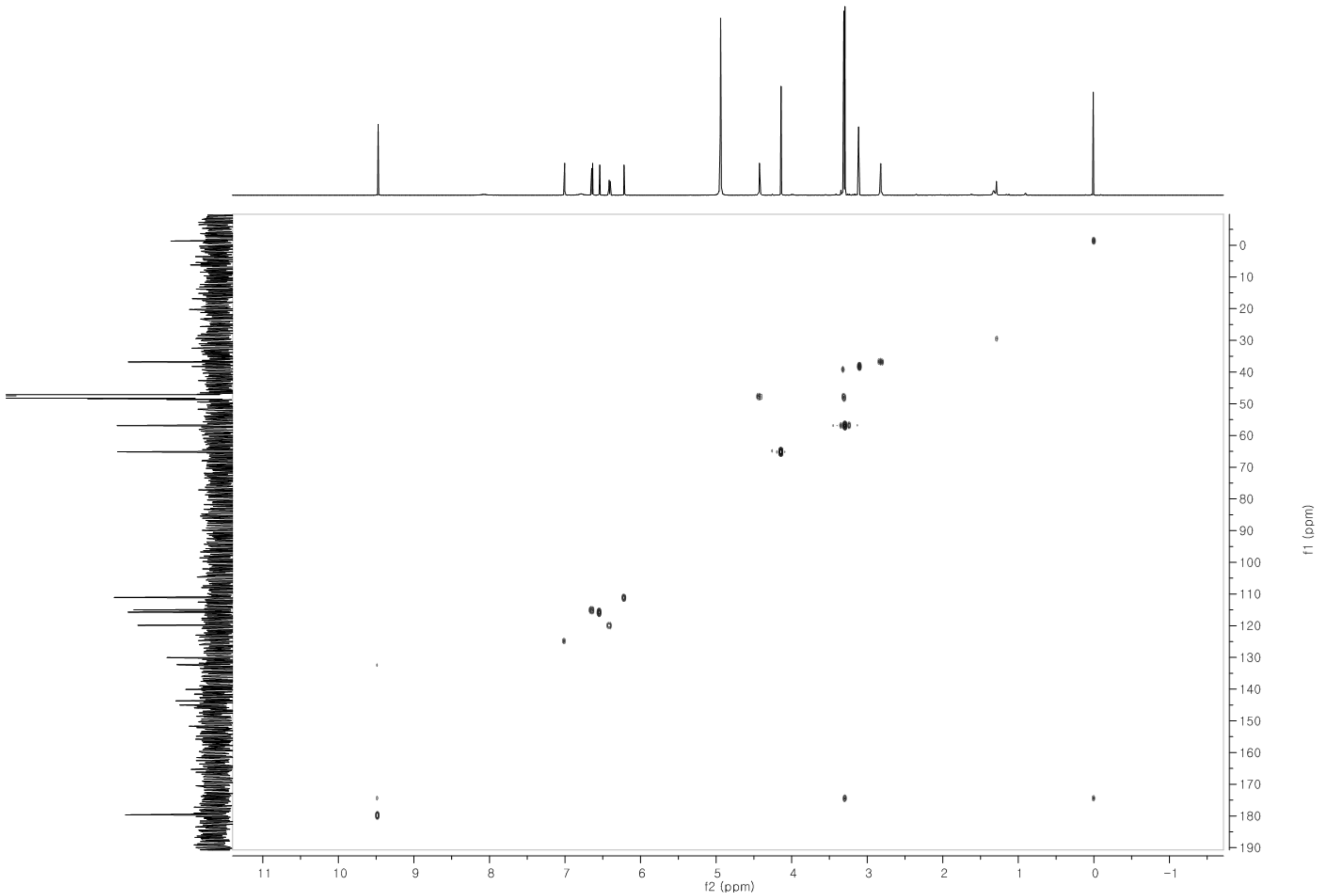

Figure S12. HMBC (600 MHz, methanol-d_4) spectrum of compound **15**

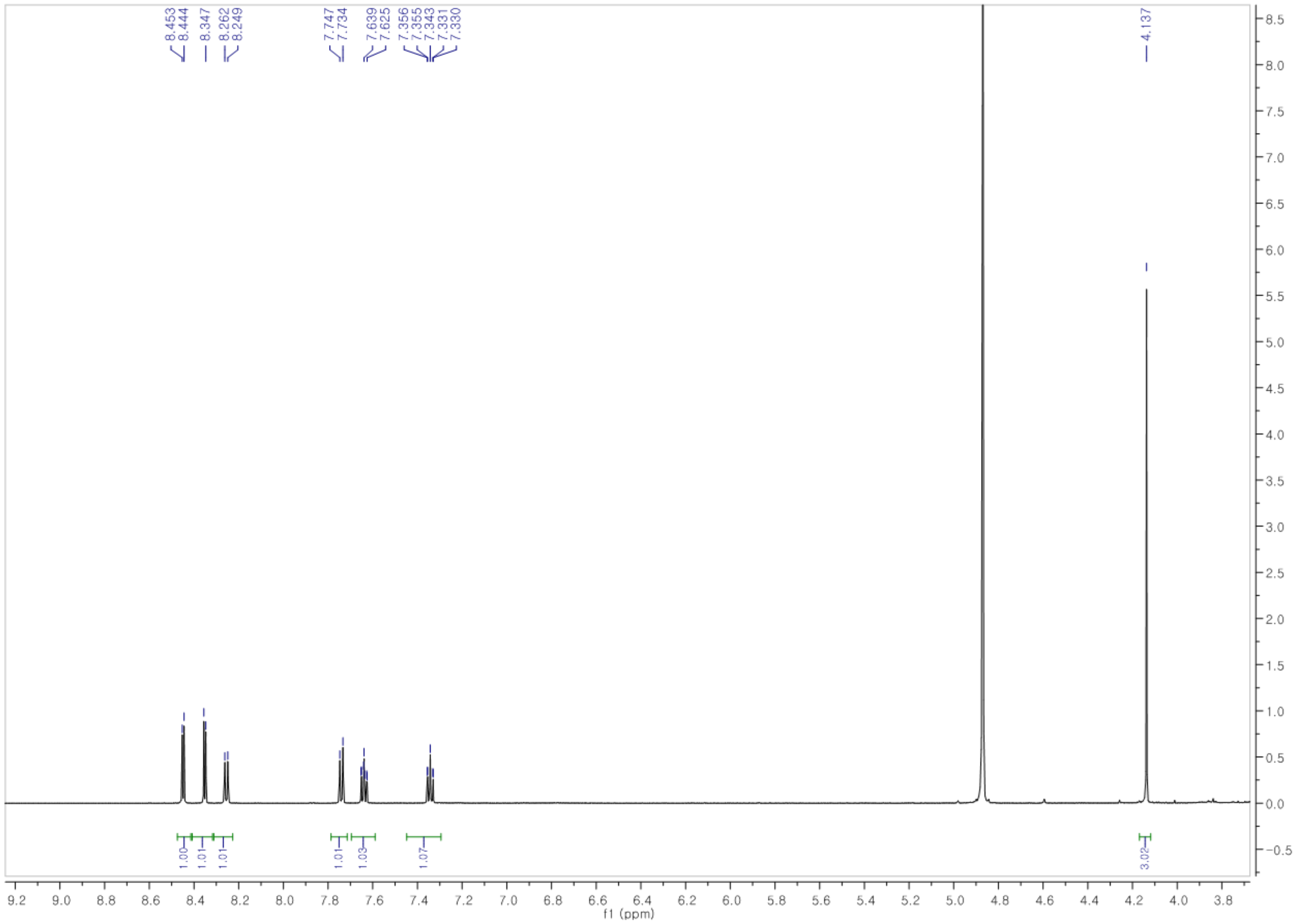

Figure S13. ^{1}H NMR (600 MHz, methanol-d_4) spectrum of compound **20**

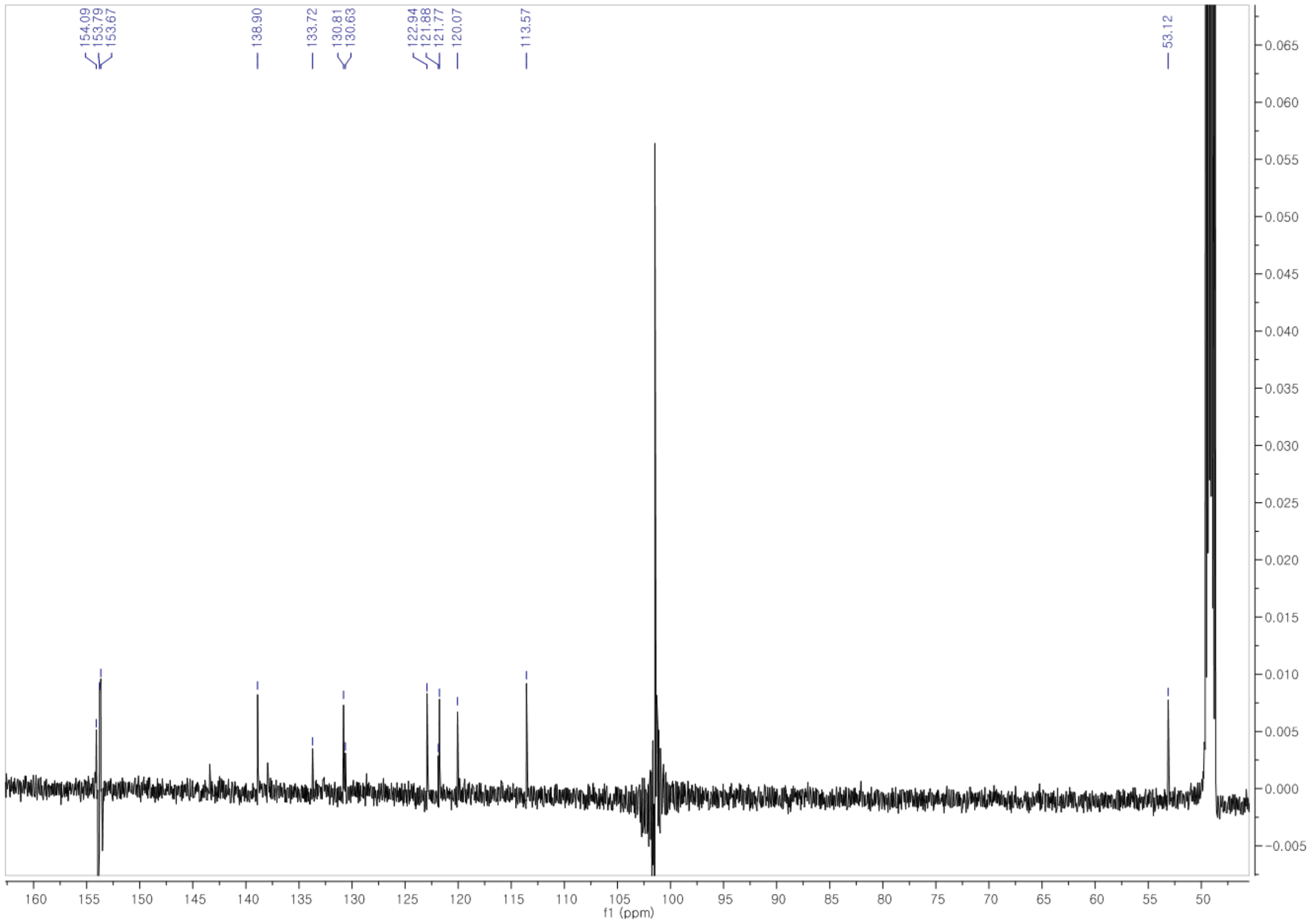

Figure S14. ^{13}C NMR (150 MHz, methanol-d_4) spectrum of compound **20**

Compound **1**: White solid; $[\alpha]_D^{25}$ = – 40 (*c* 0.1, MeOH); ESI-MS *m/z* 284.0 $[M+H]^+$; ^1H NMR (methanol-d_4, 600 MHz) δ_H 7.44 (1H, d, *J* = 15.6 Hz, H-8), 7.40 (2H, d, *J* = 8.4 Hz, H-2, 6), 7.05 (2H, d, *J* = 8.4 Hz, H-2' , 6'), 6.79 (2H, d, *J* = 8.4 Hz, H-3, 5), 6.72 (2H, d, *J* = 8.4 Hz, H-3', 5'), 6.38 (1H, d, *J* = 15.6 Hz, H-7), 3.46 (2H, t, *J* = 7.8 Hz, H-8'), 2.75 (2H, t, *J* = 7.8 Hz, H-7'); ^{13}C NMR (methanol-d_4, 150 MHz) δ_C 169.4 (C-9), 160.7 (C-4), 157.1 (C-4'), 141.9 (C-7), 131.7 (C-1'), 130.9 (C-2', 6'), 130.7 (C-2, 6), 127.9 (C-1), 118.6 (C-8), 116.9 (C-3', 5'), 116.4 (C-3, 5), 42.7 (C-8'), 36.0 (C-7').

Compound **2**: Yellowish oil; $[\alpha]_D^{25}$ = – 11 (*c* 0.1, MeOH); ESI-MS *m/z* 298.1 $[M-H]^+$; ^1H NMR (methanol-d_4, 600 MHz) δ_H 7.43 (1H, d, *J* = 15.6 Hz, H-7), 7.38 (2H, d, *J* = 8.4 Hz, H-2, 6), 7.20 (2H, d, *J* = 8.4 Hz, H-2' ,6'), 6.76 (2H, d, *J* = 8.4 Hz, H-3, 5), 6.75 (2H, d, *J* = 8.4 Hz, H-3', 5'), 6.43 (1H, d, *J* =15.6 Hz, H-8), 4.69 (1H, dd, *J* = 4.8, 4.8 Hz, H-7'), 3.51 (1H, dd, *J* = 4.8, 14.4 Hz, H-8'a), 3.42 (1H, dd, *J* = 4.8, 14.4 Hz, H-8'b); ^{13}C NMR (methanol-d_4, 150 MHz) δ_C 169.7 (C-9), 160.7 (C-4), 158.3 (C-4'), 142.1 (C-7), 134.9 (C-1), 130.7 (C-8), 128.6 (C-2, 6), 127.9 (C-2', 6'), 118.5 (C-8), 116.9 (C-3, 5), 116.3 (C-3', 5'), 73.6 (C-7'), 30.9 (C-8').

Compound **3**: Dark brown oil; $[\alpha]_D^{25}$ = – 9 (*c* 0.1, MeOH); ESI-MS *m/z* 314.1 $[M+H]^+$; ^1H NMR (methanol-d_4, 600 MHz) δ_H 7.45 (1H, d, *J* = 15.6 Hz, H-7), 7.13 (1H, d, *J* = 1.8 Hz, H-2), 7.07 (2H, d, *J* = 8.4 Hz, H-2', 6'), 7.04 (1H, dd, *J* = 8.4, 1.8 Hz, H-6), 6.81 (d, 1H, *J* = 8.4 Hz, H-5), 6.73 (2H, d, *J* = 8.4 Hz, H-3', 5'), 6.42 (1H, d, *J* = 15.6 Hz, H-8), 3.90 (3H, s, OCH_3-3), 3.48 (2H, t, *J* = 7.2 Hz, H-8), 2.77(2H, t, *J* = 7.2 Hz, H-7); ^{13}C NMR (methanol-d_4, 150 MHz) δ_C 169.3 (C-9), 157.1 (C-4'), 150.0 (C-4), 149.4 (C-3), 142.1 (C-7), 131.4 (C-1'), 130.9 (C-2'), 129.3 (C-6'), 128.4 (C-1), 123.3 (C-6), 118.9 (C-8), 116.6 (C-5), 116.4 (C-3', 5'), 111.7 (C-2), 56.5 (OCH_3-3), 42.7 (C-8'), 36.0 (C-7').

Compound **4**: Yellowish oil; $[\alpha]_D^{25}$ = – 7 (*c* 0.1, MeOH); ESI-MS *m/z* 327.9 $[M-H]^+$; ^1H NMR (methanol-d_4, 600 MHz) δ_H 7.46 (1H, d, *J* = 15.6 Hz, H-7), 7.24 (2H, d, *J* = 8.4 Hz, H-2', 6'), 7.14 (1H, d, *J* = 1.8 Hz, H-5), 7.05 (1H, dd, *J* = 8.4, 1.8 Hz, H-6), 6.82 (1H, d, *J* = 8.4 Hz, H-2), 6.79 (2H, d, *J* = 8.4Hz, H-3', 5'), 6.48 (1H, d, *J* = 15.6 Hz, H-8), 4.74 (1H, dd, *J* = 7.8, 4.8 Hz, H-7'), 3.90 (3H, s, OCH_3-3), 3.55 (1H, dd, *J* = 13.8, 4.8 Hz, H-8'b), 3.46 (1H, dd, *J* = 13.8, 7.8 Hz, H-8'a); ^{13}C NMR (methanol-d_4, 150 MHz) δ_C 169.6 (C-9), 158.2 (C-4), 150.0 (C-4'), 149.4 (C-3), 142.4 (C-7), 134.9 (C-1), 128.6 (C-2', 6'), 128.4 (C-1'), 123.4 (C-8), 118.8 (C-6), 116.6 (C-5), 116.2 (C-3', 5'), 111.7 (C-2), 73.6 (C-7'), 56.5 (OCH_3-3), 48.5 (C-8').

Compound **5**: Yellowish oil; $[\alpha]_D^{25}$ = – 15 (*c* 0.1, MeOH); ESI-MS *m/z* 344.0 $[M+H]^+$; ^1H NMR (methanol-d_4, 600 MHz) δ_H 7.46 (1H, d, *J* = 15.6 Hz, H-7), 7.19 (2H, d, *J* = 9.0 Hz, H-2', 6'), 7.14 (1H, d, *J* =

1.8 Hz, H-5), 7.04 (1H, dd, *J* = 8.4, 1.8 Hz, H-6), 6.82 (1H, d, *J* = 8.4, 1.8 Hz, H-3', 5'), 6.49 (1H, d, *J* = 15.6 Hz, H-8), 4.26 (1H, dd, *J* = 8.4, 4.2 Hz, H-7'), 3.90 (3H, s, OCH_3-3), 3.53 (1H, dd, *J* = 13.8, 4.8 Hz, H-8'b), 3.42 (1H, dd, *J* = 13.8, 9.0 Hz, H-8'a), 3.23 (3H, s, OCH_3-7'); ^{13}C NMR (methanol-d_4, 150 MHz) δ_C 169.3 (C-9), 158.7 (C-4), 150.0 (C-4'), 149.4 (C-3), 142.3 (C-7), 131.6 (C-1), 129.3 (C-2', 6'), 128.4 (C-1'), 123.4 (C-8), 118.8 (C-6), 116.6 (C-5), 116.5 (C-3', 5'), 111.7 (C-2), 83.4 (C-7'), 57.0 (OCH_3-3), 56.5 (OCH_3-7'), 47.2 (C-8').

Compound **6**: Dark brown oil; $[\alpha]_D^{25}$ = – 17 (*c* 0.1, MeOH); ESI-MS *m*/*z* 374.0 $[M+H]^+$; ^1H NMR (methanol-d_4, 600 MHz) δ_H 7.46 (1H, d, *J* = 15.6 Hz, H-7), 7.13 (1H, d, *J* = 1.8 Hz, H-2), 7.04 (1H, dd, *J* = 13.8, 1.8 Hz, H-6), 6.83 (1H, d, *J* = 1.8 Hz, H-2'), 6.81 (1H, d, *J* = 7.8 Hz, H-5), 6.74 (1H, d, *J* = 8.4 Hz, H-5'), 6.68 (1H, dd, *J* = 8.4, 1.8 Hz, H-6'), 6.43 (1H, d, *J* = 15.6 Hz, H-8), 3.89 (3H, s, OCH_3-3), 3.85 (3H, s, OCH_3-3'), 3.50 (2H, t, *J* = 7.2 Hz, H-8'), 2.78 (2H, t, *J* = 7.2 Hz, H-7'); ^{13}C NMR (methanol-d_4, 150 MHz) δ_C 169.3 (C-9), 150.0 (C-4), 149.4 (C-3), 149.1 (C-3'), 146.2 (C-4'), 142.2 (C-7), 132.2 (C-1'), 128.4 (C-1), 123.3 (C-6), 122.4 (C-6'), 118.9 (C-8), 116.3 (C-5), 113.6 (C-2), 111.7 (C-2'), 56.5 (OCH_3-3), 56.5 (OCH_3-3'), 42.6 (C-8'), 36.4 (C-7').

Compound 7: Yellowish oil; $[\alpha]_D^{25}$ = – 20 (*c* 0.1, MeOH); ESI-MS *m*/*z* 357.9 $[M-H]^+$; ^1H NMR (methanol-d_4, 600 MHz) δ_H 7.46 (1H, d, *J* = 15.6 Hz, H-7), 7.15 (1H, d, *J* = 1.8 Hz, H-2), 7.05 (1H, dd, *J* = 7.8, 1.8 Hz, H-6), 7.02 (1H, d, *J* = 1.8 Hz, H-2'), 6.85 (1H, dd, *J* = 8.4, 1.8 Hz, H-5), 6.82 (1H, d, *J* = 8.4 Hz, H-5'), 6.80 (1H, d, *J* = 7.8 Hz, H-6'), 6.49 (1H, d, *J* = 15.6 Hz, H-8), 4.75 (1H, dd, *J* = 7.8, 4.8 Hz, H-7'), 3.90 (3H, s, OCH_3-3), 3.88 (3H, s, OCH_3-3'), 3.57 (1H, dd, *J* = 13.8, 4.8 Hz, H-8'b), 3.47 (1H, dd, J = 13.8, 7.8 Hz, H-8'a); ^{13}C NMR (methanol-d_4, 150 MHz) : δ_C 169.6 (C-9), 150.0 (C-4), 149.4 (C-3'), 149.1 (C-3), 147.3 (C-4'), 142.4 (C-7), 135.6 (C-1'), 128.4 (C-1), 123.4 (C-6), 120.1 (C-6'), 118.8 (C-8), 116.6 (C-5), 116.1 (C-5'), 111.7 (C-2), 111.0 (C-2'), 73.8 (C-7'), 56.5 (OCH_3-3), 56.50 (OCH_3-3'), 48.51 (C-8').

Compound **8**: White solid; $[\alpha]_D^{25}$= – 18 (*c* 0.1, MeOH); HRESIMS *m*/*z* 396.1422 $[M+Na]^+$ (396.1423, calcd for $C_{20}H_{23}O_6Na$).

Compound **9**: Dark brown oil; $[\alpha]_D^{25}$ = – 7 (*c* 0.1, MeOH); ESI-MS *m*/*z* 374.1 $[M+H]^+$; ^1H NMR (methanol-d_4, 600 MHz) δ_H 7.47 (1H, d, *J* = 15.6 Hz, H-7), 7.13 (1H, d, *J* = 1.8 Hz, H-2), 7.05 (1H, d, *J* = 7.8, 1.8 Hz, H-6), 6.82 (1H, d, *J* = 8.4 Hz, H-5), 6.54 (2H, s, H-2', H-6'), 6.44 (1H, d, *J* = 15.6 Hz, H-8), 3.90 (3H, s, OCH_3-3), 3.84 (3H, s, OCH_3-3', OCH_3-5'), 3.52 (2H, t, *J* = 7.2 Hz, H-8'), 2.80 (2H, t, *J* = 7.2 Hz, H-7'); ^{13}C NMR (methanol-d_4, 150 MHz) δ_C 169.3 (C-9), 150.0 (C-3'), 149.4 (C-5'), 149.4 (C-3, 4), 142.2 (C-7), 135.2

(C-4'), 131.4 (C-1'), 128.4 (C-1), 123.3 (C-6), 118.9 (C-8), 116.6 (C-5), 111.7 (C-2), 107.2 (C-6'), 105.12 (C-2'),56.9 (OCH_3-3, OCH_3-3'), 56.5 (OCH_3-5'), 42.6 (C-8'), 36.8 (C-7').

Compound **10**: Colorless gum; $[\alpha]_D^{25}$ = + 27 (*c* 0.1, MeOH); ESI-MS *m/z* 344.1 $[M+H]^+$; ^1H NMR (methanol-d_4, 600 MHz) δ_H 7.39 (1H, d, *J* = 2.4 Hz, H-2), 7.14 (2H, d, *J* = 8.4 Hz, H-2', 6'), 6.97 (1H, d, *J* = 14.4, 1.8 Hz, H-6), 6.78 (2H, d, *J* = 9.0 Hz, H-5), 6.76 (1H, d, *J* = 8.4 Hz, H-3', 5'), 6.65 (1H, d, *J* = 12.6 Hz, H-7), 5.85 (1H, d, *J* = 12.6 Hz, H-8), 4.18 (1H, dd, *J* = 8.4, 4.2 Hz, H-7'), 3.86 (3H, s, OCH_3-3), 3.46 (1H, dd, *J* = 13.8, 4.2 Hz, H-8'), 3.15 (3H, s, OCH_3-7'); ^{13}C NMR (methanol-d_4, 150 MHz) δ_C 170.3 (C-9), 158.7 (C-4'), 148.7 (C-4), 148.6 (C-3), 138.8 (C-7), 131.4 (C-1'), 129.3 (C-2', 6'), 128.6 (C-1), 125.0 (C-6), 121.7 (C-8), 116.5 (C-3', 5'), 116.0 (C-5), 114.2 (C-2), 83.2 (C-7'), 56.9 (OCH_3-7'), 56.5 (OCH_3-3), 47.0 (C-8').

Compound **11**: Pale Yellow needles; $[\alpha]_D^{25}$ = – 43 (*c* 0.1, MeOH); ESI-MS *m/z* 445.3 $[M+H]^+$; ^1H NMR (DMSO-d_6, 600 MHz) δ_H 8.51 (1H, d, *J* = 8.4 Hz, N-Hb), 8.14 (1H, d, *J* = 8.4 Hz, N-Ha), 7.78 (2H, d, *J* = 7.2 Hz, H-16, 20), 7.50 (1H, t, *J* = 7.2 Hz, H-18), 7.43 (2H, t, *J* = 8.4 Hz, H-17, 19), 7.31 (2H, d, *J* = 8.4 Hz, H-23, 27), 7.22 (6H, m, H-5, 7, 9, 24, 25, 26), 7.15 (2H, m, H-6, 8), 4.67 (1H, m, *J* = 4.8 Hz, H-13), 4.18 (1H, m, *J* = 5.4 Hz, H-2), 4.01 (1H, dd, *J* = 10.8, 4.8 Hz, H-10b), 3.85 (1H, dd, *J* = 10.8, 4.8 Hz, H-10a), 2.97 (2H, m, H-21), 2.78 (2H, m, H-3), 1.97 (3H, s, H-12); ^{13}C NMR (DMSO-d_6, 150 MHz) δ_C 171.2 (C-11), 170.2 (C-1), 166.1 (C-14), 138.3 (C-22), 138.0 (C-4), 134.0 (C-15), 131.3 (C-18), 129.1 (C-6, 8), 128.2 (C-5, 9), 128.1 (C-17, 19), 128.0 (C-25), 127.4 (C-16, 20), 126.2 (C-7), 64.6 (C-10), 54.9 (C-13), 49.1 (C-2), 37.2 (C-21), 36.6 (C-3), 20.6 (C-12).

Compound **12**: Amorphous powder; $[\alpha]_D^{25}$ = + 8 (*c* 0.1, MeOH); ESI-MS *m/z* 222.1 $[M+H]^+$; ^1H NMR (methanol-d_4, 600 MHz) δ_H 7.27 (2H, t, 7.2 Hz, H-3, 5), δ_H 7.23 (3H, m, H-2, 4, 6), 4.67 (1H, dd, *J* = 8.4, 6.0 Hz, H-8), 3.70 (3H, s, OCH_3-9), 3.16 (1H, dd, *J* = 13.8, 6.0 Hz, H-7a), 2.97 (1H, dd, *J* = 13.8, 9.0 Hz, H-7b), 1.92 (3H, s, H-11); ^{13}C NMR (methanol-d_4, 150 MHz) δ_C 173.7 (C-9), 173.3 (C-10), 138.3 (C-1), 130.3 (C-2, 6), 129.6 (C-3, 5), 128.0 (C-4, 55.5 (C-8), 52.8 (OCH_3-9), 38.6 (C-7), 22.4 (C-11).

Compound **13**: Pale Yellow needles; $[\alpha]_D^{25}$ = – 5 (*c* 0.1, MeOH); ESI-MS *m/z* 266.0 $[M+H]^+$; ^1H NMR (methanol-d_4, 600 MHz) δ_H 7.84 (2H, dd, 7.2, 1.2 Hz, H-2', 6'), 7.58 (1H, t, 7.2 Hz, H-4'), 7.49 (2H, t, 7.8 Hz, H-3', 5'), 5.01 (1H, dd, *J* = 7.2, 6.0 Hz, H-2), 3.79 (3H, s, OCH_3-1), 3.72 (3H, s, OCH_3-4), 3.06 (1H, dd, *J* = 16.8, 6.0 Hz, H-3a), 2.94 (1H, dd, *J* = 16.8, 7.2 Hz, H-3b); ^{13}C NMR (methanol-d_4, 150 MHz) δ_C 172.9 (C-1), 172.7 (C-4), 170.2 (C-7'), 135.1 (C-1'), 133.2 (C-4'), 129.7 (C-2', 6'), 128.6 (C-3', 5'), 53.3 (OCH_3-1), 52.6

(OCH_3-4), 51.1 (C-2), 36.8 (C-3).

Compound **14**: Pale Yellow needles; $[\alpha]_D^{25}$ = + 6 (*c* 0.1, MeOH); ESI-MS: *m/z* 266.0 $[M+H]^+$; ^{1}H NMR (methanol-d_4, 600 MHz) δ_H 7.84 (2H, dd, *J* = 7.2, 1.2 Hz, H-2', 6'), 7.58 (1H, t, *J* = 7.2 Hz, H-4'), 7.49 (2H, t, *J* = 7.8 Hz, H-3', 5'), 5.01 (1H, dd, *J* = 7.2, 6.0 Hz, H-2), 3.79 (3H, s, OCH_3-1), 3.72 (3H, s, OCH_3-4), 3.06 (1H, dd, *J* = 10.8, 6.0 Hz, H-3a), 2.94 (1H, dd, *J* = 10.8, 4.8 Hz, H-3b); ^{13}C NMR (methanol-d_4, 150 MHz) δ_C 172.9 (C-1), 172.8 (C-4), 170.2 (C-7'), 135.1 (C-1'), 133.2 (C-4'), 129.8 (C-2', 6'), 128.6 (C-3', 5'), 53.3 (OCH_3-1), 52.6 (OCH_3-4), 51.1 (C-2), 36.8 (C-3).

Compound **15**: Brown liquid; $[\alpha]_D^{25}$ = + 15 (*c* 0.1, MeOH); ESI-MS *m/z* 273.8 $[M-H]^+$.

Compound **16**: Pale white powder; $[\alpha]_D^{25}$ = + 55 (*c* 0.1, MeOH); ESI-MS *m/z* 220.0 $[M+H]^+$; ^{1}H NMR (DMSO-d_6, 600 MHz) δ_H 8.77 (1H, s, 8-OH), 8.7 (1H, s, 7-OH), 6.51 (1H, s, H-10), 6.49 (1H, s, H-7), 4.57 (1H, t, *J* = , H-10b), 3.96 (1H, dt, *J* = , H-5), 2.91 (1H, m, H-5), 2.54 (1H, m, H-1), 2.54 (2H, m, H-6), 2.40 (1H, m, H-2), 2.22 (1H, m, H-2) 1.60 (1H, m, H-1); ^{13}C NMR (DMSO-d_6, 150 MHz) δ_C 171.9 (C-3), 144.1 (C-9), 143.9 (C-8), 128.3 (C-10a), 123.6 (C-6a), 115.3 (C-7), 111.6 (C-10), 55.5 (C-10b), 36.6 (C-5), 31.2 (C-2), 27.3 (C-6), 27.3 (C-1).

Compound **17**: White powder; $[\alpha]_D^{25}$ = + 24 (*c* 0.1, MeOH); ESI-MS *m/z* 198.1 $[M+H]^+$; ^{1}H NMR (DMSO-d_6, 600 MHz) δ_H 3.68 (3H, s, OCH_3-3'), 2.70 (2H, m, H-1'), 2.63 (2H, m, H-2'), 1.97 (3H, s, CH_3-4); ^{13}C NMR (DMSO-d_6, 150 MHz) δ_C 174.5 (C-3'), 174.5 (C-5), 174.3 (C-2), 140.8 (C-3), 140.4 (C-4), 52.4 (OCH_3-3'), 32.8 (C-2'), 20.2 (C-1'), 8.6 (CH_3-4).

Compound **18**: Pink solid; $[\alpha]_D^{25}$ = + 22 (*c* 0.1, MeOH); ESI-MS *m/z* 146.1 $[M+H]^+$; ^{1}H NMR (DMSO-d_6, 600 MHz) δ_H δ 9.91 (1H, s, H-10), 8.18 (1H, d, *J* = 7.8 Hz, H-4), 8.12 (1H, s, H-2), 7.50 (1H, d, *J* = 7.8 Hz, H-7), 7.29 (1H, td, *J* = 7.2, 1.2 Hz , H-5), 7.25 (1H, td, *J* = 7.2, 1.2 Hz, H-6); ^{13}C NMR (DMSO-d_6, 150 MHz) δ_C 187.5 (C-10), 139.9 (C-2), 139.8 (C-8), 125.9 (C-9), 125.1 (C-4), 123.8 (C-6), 122.5 (C-5), 120.3 (C-3), 113.3 (C-7).

Compound **19**: White powder; $[\alpha]_D^{25}$ = + 32 (*c* 0.1, MeOH); ESI-MS *m/z* 174.1 $[M+H]^+$; ^{1}H NMR (methanol-d_4, 600 MHz) δ_H 8.05 (1H, m, H-4), 7.95 (1H, s, H-2), 7.44 (1H, m, H-7), 7.22 (2H, m, H-5, 6), 4.36 (2H, dd, *J* = 14.4, 7.2 Hz, H-11a, 11b), 1.41 (3H, t, *J* = 10.8 Hz, H-12); ^{13}C NMR (methanol-d_4, 150 MHz) δ_C 167.7 (C-10), 138.3 (C-2), 133.3 (C-8), 127.4 (C-9), 123.8 (C-4), 122.6 (C-6), 122.1 (C-5), 113.1 (C-3), 108.7 (C-7), 60.9 (C-11), 15.0 (C-12).

Compound **20**: Colorless needles; $[\alpha]_D^{25}$ = + 20 (*c* 0.1, MeOH); ESI-MS *m/z* 227.0 $[M+H]^+$.

Compound **21**: amorphous powder; $[\alpha]_D^{25}$ = + 17 (*c* 0.1, MeOH); ESI-MS *m/z* 144.1 $[M+H]^+$; ^1H NMR (methanol-d_4, 600 MHz) δ_H 4.35 (1H, dd, *J* = 9.6, 4.8 Hz, H-3), 3.81 (3H, s, OCH_3-6), 2.57 – 2.19 (4H, m, H-4, 5); ^{13}C-NMR (methanol-d_4, 150 MHz) δ_C 181.1 (C-1), 174.6 (C-6), 57.1 (OCH_3-6), 53.0 (C-3), 30.4 (C-5), 26.0 (C-4).

Compound **22**: Colorless amorphous powder; $[\alpha]_D^{25}$ = + 35 (*c* 0.1, MeOH); ESI-MS *m/z* 158.1 $[M+H]^+$; ^1H NMR (methanol-d_4, 600 MHz) δ_H 4.32 (1H, dd, *J* = 9.0, 4.2 Hz, H-3), 4.25 (2H, q, *J* = 7.2 Hz, H-7), 2.56 – 2.17 (4H, m, H-4, 5), 1.33 (3H, s, H-8); ^{13}C NMR (methanol-d_4, 150 MHz) δ_C 181.1 (C-6), 174.1 (C-1), 62.7 (C-7), 57.3 (C-3), 30.6 (C-5), 26.0 (C-4), 14.6 (C-8).
